# Context matters: Leveraging anthropology within one health

**DOI:** 10.1016/j.onehlt.2022.100393

**Published:** 2022-05-02

**Authors:** Travis S. Steffens, Elizabeth Finnis

**Affiliations:** a Department of Sociology and Anthropology, University of Guelph, Guelph, Ontario, Canada; b Planet Madagascar, Guelph, Ontario, Canada

**Keywords:** Community-based, Long-term, Conservation, India, Madagascar, Ground-up

## Abstract

Anthropologists develop long-term engagements with communities, animals, and the ecosystems they all share. This approach can provide important context that is necessary for One Health research, which may otherwise overlook the perspectives and lived experiences of community members. This paper presents two case studies that illustrate the importance of leveraging long-term, holistic, engagements with communities in moving the One Health concept forward. The first illustrates the complexity of understanding the health of people and animals within the context of environmental change in South India. The second provides insights into how the conservation of endangered species requires considering the entanglements of people, domestic animals, and the landscapes they share with wildlife in Madagascar. We demonstrate the value of integrating anthropological perspectives within interdisciplinary One Health research and interventions to better understand the complexity of systems.

## Highlights

• One Health research should incorporate anthropologists and/or social scientists.
• Anthropology provides unique approaches to improve One Health research.
• Long-term engagements with communities will improve One Health research.

## 1 Introduction

One Health in part has its roots in a collaboration between veterinary medicine and public health, and as such the One Health approach has been primarily focussed on zoonotic disease [1]. One Health collaborations across academic and non-academic/policy sectors can result in practical outcomes such as shared priorities for the development of health monitoring and response programs [2,3]. However, if collaborations do not integrate community-based, lived-experience understandings of health and disease, they risk being anthropocentric and/or ethnocentric, and may provide only partial insights into the range of issues that can be approached from a One Health perspective. As such, anthropological methodologies and theoretical approaches provide important context necessary to moving One Health agendas forward. Here, we add to the literature that calls for the centering of anthropology and related social sciences to ensure robust One Health programs [e.g., [[4], [5], [6]]], by presenting two case studies that illustrate the importance of leveraging long-term, holistic, engagements with communities.

Anthropologists have a long history of conducting research that addresses core aspects of the One Health approach, even though not labelled as such. There is a range of anthropological research that considers and theorizes relationships among humans, animals, and the landscape in diverse and sometimes shifting cultural, political, and/or climate contexts [e.g., [[7], [8], [9], [10], [11], [12]]]. And, biocultural approaches recognize the interactions between biological processes and historical, political, economic, and cultural contexts [[13], [14], [15], [16]], contextualizing notions of health, ill-health, and risk within specific cultural and environmental relations [e.g., [4,13,17,18]. This creates opportunities to develop understandings of what Lock [19] has referred to as local biologies, which reflect the nuances and specificities of the ways that local conceptualizations of health and wellbeing can go beyond biomedical assessments to encompass emotional, spiritual, environmental, or other elements [20]. Thus, ground-up approaches are necessary to guide One Health research questions and projects. Our case illustrations emerge from long-term anthropological work in India and Madagascar and demonstrate 1) How understandings of intersections among human, ecosystem, and animal health can emerge, sometimes in unanticipated ways, and beyond a focus on zoonoses; and 2) The potential that long-term, community engagement has for the development of One Health projects that account for experiences and perceptions of the multiple dimensions of health.

Illustration 1: The Kolli Hills, Tamil Nadu, India.

This case emerges from ethnographic research and highlights the ways perceptions of health can be tied to food and environmental changes. Between 2003 and 2006, and with a follow-up visit in 2010, Finnis worked with smallholder farmers in four neighbouring villages with a focus on changing livelihood and dietary patterns. Methods included semi-structured interviews and focus groups on environment, labour, agricultural decision-making, and food access, dietary recalls, participatory mapping, and participant observation. Participant observation, a core anthropological method, entails living and working with people over long periods of time, situating research within everyday life.

Changing and unpredictable rainfall patterns meant that traditional millet subsistence crops could no longer be reliably grown. Instead, farmers had come to depend on cassava, a cash crop that generated income used for food, household goods, and education [21]. Instead of eating millets, farmers were largely reliant on purchased rice. The cultural values regarding rice were complex. When community members had relied on millets for everyday meals, rice was seen as a higher-status and luxury food, symbolizing the ability to buy, rather than grow, food. Purchased rice is also faster to prepare and cook than millets. However, as millets became increasingly rare, their loss manifested in discussions of ill health for community members and their livestock. Millets were described as giving strength, filling the stomach and preventing hunger, and contributing to overall health and robustness, characteristics that were reflected in the nutritional value of relying on diverse core grains [22]. Farmers argued that eating rice was less healthy, but they had no other options. The notion of dryness emerged in discussions, connecting people's concerns about rainfall pattern changes and food crops with their own bodies. Without seasonal rains, millets were at risk of withering or becoming “dry,” before they were ready for harvest. As one woman put it, their bodies felt like “dry husks.”

Moreover, as cassava yields decreased over time, farmers expanded fields by clearing forest land and moving into pastureland. Community members mourned the loss of forests, a process described as tragic but that they felt largely helpless to address. The loss of pastureland meant livestock were less well fed and more at risk of disease. With no easily accessible veterinary care options, owning larger numbers of livestock was an increasingly risky endeavour for both people and animals. Essentially, degradation of the local ecosystem and resulting food changes became mapped onto local human and animal bodies, contributing to an overall sense of ill health and of loss that was not just about nutrition, but also about a broader sense of wellbeing that was shaped by access to culturally valued foods, spaces, and practices. By considering the richness of localized perceptions of human-environment-animal connections we see how One Health ideas are not academic constructs alone; rather, they emerge in the everyday lived experiences of people who do not necessarily draw distinctions among individual health, the health of their environment, and the health of livestock [20].

Illustration 2: Ankarafantsika National Park, Madagascar.

This case began as a conservation biogeography research project on lemurs in 2010 which transformed into a holistic research, conservation, and community development program in 2015 in Ambanjabe Field Site (AFS) within Ankarafantsika National Park, Madagascar. Within AFS (8000 ha) there are three Sakalava communities along a river valley of gallery forest adjacent to a fragmented landscape of dry deciduous forest and anthropogenic grassland [23]. People in these communities live a mostly subsistence agricultural lifestyle centred on growing rice and raising Zebu cattle. Rice (*vary* in Malagasy) and zebu (*Bos taurus indicus*) are important cultural touchstones that shape conceptions of being healthy. “Rice is life” is a common proverb among Malagasy and a day without rice is “empty”. Zebu cattle serve important roles in ceremony and are meaningful in ways beyond simply food [24]. Additionally, the park itself holds meaning because it contains many spiritual sites, where residents can commune with ancestors [25]. Thus, how people conceive their relationship between themselves, their ancestors, lemurs, and the forests they all share have important health, wellbeing, and conservation implications.

Recognizing the connection between people, their domestic animals, forests, and lemurs, Steffens began a community-focussed conservation, development, and education program through a NGO, Planet Madagascar, to build capacity to create sustainable forest communities. Engagements with community members demonstrated that previous attempts to improve income and food security had forced decisions that had negative ecological and health consequences for people, zebu, and lemurs. For example, the main cause of habitat change in the forest near each community was fire set by “outsiders”, hired by wealthy zebu owners who lived outside the park, to promote new grass growth for zebu to graze on. These fires contributed to decreased forest and increased erosion affecting nearby rice fields [26] and their capacity to produce food. During the grazing season these “outsiders”, themselves reliant on the forest nearby the grazing lands, degraded the forest and affected lemurs by collecting non-forest timber products, cutting trees, and hunting. Residents have little power to stop “outsiders” from using forest adjacent to their communities and personal security is a major concern for them. Residents are constrained by national park rules and are not allowed to increase their agricultural production – forcing them to move further from their community to improve income and food security – becoming the “outsiders” to another community. Zebu and lemurs are stuck in the middle of the clash between residents and “outsiders” needs while all are impacted by degraded and decreased forest habitat. Therefore, any attempt to directly address conservation issues affecting lemurs requires communicating with residents, zebu owners and hired workers, and the communities where the residents become the “outsiders”. It is also important to consider and take seriously local conceptualizations of health and wellness, as well as their perceptions towards lemurs, forests, and conservation. These considerations have led Steffens to develop an emerging One Health program, which draws on long-term relationships and in-depth understandings of the cultural, environmental, economic, and political factors that shape the interface among people, animals, and the environment. Using this ground-up approach to understand and address complex scenarios will provide more nuanced results reflecting local contexts.

## 2 Putting community at the centre of research

Inter-, multi-, or transdisciplinary collaborations are critical to the development, functioning, and success of One Health initiatives in diverse social, cultural, geographical, multispecies, and health contexts. Anthropological research methods that prioritize long-term engagements with communities (human and non-human) and draw on a combination of qualitative, quantitative, and participatory research methods, offer depth to diverse One Health initiatives, filling gaps in knowledge and engagement [4,6,27,28]. The inclusion of highly localized knowledge can lead to more holistic One Health approaches, including around key knowledge gaps and strengths [29] and emerging risks [30]. This creates space within any community for the integration of different types of knowledge, and for “knowledge co-creation” [31] through participatory approaches [32]. Privileging local voices and experiences allows for the development of community-based understandings of how health risks play out and are conceived, perceived, and/or experienced at highly localized levels [27,33], in the process going beyond simply involving communities in pre-established academic or policy frameworks [34].

These case illustrations demonstrate the value of anthropological approaches in determining and contextualizing local understandings of health and wellbeing, and local perspectives on human-animal-environmental health intersections. And, as demonstrated, even everyday staple foods like rice may have very different meanings when viewed through the specific cultural lenses that shape concepts of health and wellbeing. Rice in the Kolli Hills, India, is conceptualized as both a food signaling economic capacity, but also as less nutritious, less filling, and less culturally valuable than traditional millet varieties. In AFS, Madagascar “rice is life” and a meal without rice is unsatisfying and contributes to a decreased sense of wellbeing. While more nutrient dense foods including other grains are available, the Sakalava prefer to consume rice, often referring to other grains as *vary vazaha* (foreigner rice). Thus, taking the time to understand nuances and local meanings seriously contributes to potential outcome sustainability [31]; overlooking community voices may weaken and/or limit community engagement with proposed programs and interventions [20]. We also demonstrate that there are practical program implications and potential theoretical benefits of expanding One Health approaches and concepts beyond considerations of infectious diseases. We therefore urge 1) One Health researchers based in the veterinary and biological sciences to collaborate with anthropologists and other social scientists in building more holistic research agendas, and 2) anthropologists to develop and enact One Health projects that in turn leverage inter-multi-transdisciplinary approaches that involve non-social scientists.

## CRediT authorship contribution statement

**Travis S. Steffens:** Conceptualization, Writing – original draft, Writing – review & editing. **Elizabeth Finnis:** Conceptualization, Writing – original draft, Writing – review & editing.

## Declaration of Competing Interest

The authors declare no conflicts of interest. Informed consent was given by all participants in this research.
